# A two-step pre-processing tool to remove Gaussian and ectopic noise for heart rate variability analysis

**DOI:** 10.1038/s41598-022-21776-2

**Published:** 2022-11-01

**Authors:** Shiza Saleem, Ahsan H. Khandoker, Mohanad Alkhodari, Leontios J. Hadjileontiadis, Herbert F. Jelinek

**Affiliations:** 1 Department of Biomedical Engineering, Khalifa University, 127788 Abu Dhabi, United Arab Emirates; 2 Healthcare Engineering Innovation Center, Khalifa University, 127788 Abu Dhabi, United Arab Emirates; 3 Biotechnology Center, Khalifa University, 127788 Abu Dhabi, United Arab Emirates

**Keywords:** Biomedical engineering, Biomarkers, Cardiology

## Abstract

Artifacts in the Electrocardiogram (ECG) degrade the quality of the recorded signal and are not conducive to heart rate variability (HRV) analysis. The two types of noise most often found in ECG recordings are technical and physiological artifacts. Current preprocessing methods primarily attend to ectopic beats but do not consider technical issues that affect the ECG. A secondary aim of this study was to investigate the effect of increasing increments of artifacts on 24 of the most used HRV measures. A two-step preprocessing approach for denoising HRV is introduced which targets each type of noise separately. First, the technical artifacts in the ECG are eliminated by applying complete ensemble empirical mode decomposition with adaptive noise. The second step removes physiological artifacts from the HRV signal using a combination filter of single dependent rank order mean and an adaptive filtering algorithm. The performance of the two-step pre-processing tool showed a high correlation coefficient of 0.846 and RMSE value of 7.69 × 10^–5^ for 6% of added ectopic beats and 6 dB Gaussian noise. All HRV measures studied except HF peak and LF peak are significantly affected by both types of noise. Frequency measures of Total power, HF power, and LF power and fragmentation measures; PAS, PIP, and PSS are the most sensitive to both types of noise.

## Introduction

Heart rate influenced by different physiological origins such as the circadian rhythm, Mayer waves, and respiratory activity is not a constant^1,2^. This variability is described by heart rate variability (HRV), determined from the variance in time intervals between two consecutive heartbeats. HRV is assumed to be the result of rhythmic changes in the electrical activity of the heart. HRV has been shown to mirror the state of the autonomic nervous system (ANS), which regulates the cardiac activity and hence provides an insight into cardiac autonomic function^1,3–5^. The ANS has two main divisions, the parasympathetic nervous system (PNS) and the sympathetic nervous system (SNS). HRV, therefore, reflects a reciprocating sympathovagal balance between the PNS and the SNS^4,5^. An optimal HRV is a sign of a healthy heart with good modulation of the cardiac rhythm and force of contraction. Hence, decreased or an excessively increased HRV is associated with disease and can be used to assess cardiac health^6^. Over the past few decades, extensive research has shown a significant connection between ANS and cardiovascular events and supports the analysis of HRV to assess cardiovascular health^3–5,7–14^.

HRV measures are derived by applying linear and non-linear mathematical techniques to assess the changes in the time interval between R peaks (RR interval) from the Electrocardiogram (ECG) recordings. These measures can quantitatively assess HRV, but the analysis can be influenced due to the presence of artifacts. Although various measures are taken to minimize the artifacts in the recordings, signal degradation due to artifacts remains a major concern^15^. The two types of artifacts are noise in the ECG signal and physiological artifacts, ectopic beats, that lead to abnormal RR intervals (RRI) and influence HRV analysis results ^16^. ECG signals can be corrupted by noise from different sources including power line interference, baseline wander, muscle artifact, and instrumentation noise, among others. Apart from power line interference and baseline wander noise, all other sources of noise can be assumed to be of Gaussian nature^17^. Physiological artifacts include premature atrial and ventricular contractions that are a consequence of abnormal cardiac electrical conduction present as abnormal and irregular ECG rhythms^18^. Ventricular ectopic beats are observed in 40–75% of Holter recordings in normal recordings and increase in cardiac pathology and hence play an important role in HRV accuracy^19^.

There are many filtering techniques applied to denoise the ECG signal. Finite impulse response (FIR) smoothing filter, notch filter, low-pass filter, and high-pass filter are the more common ones in use. Also, Hamilton and Tompkins, or wavelet transform models are used for denoising ECGs^20^. Recent techniques widely used also include empirical mode decomposition (EMD) models and deep‐learning‐based autoencoder models (DAEs)^20–22^. Ectopic beats can be detected and denoised directly from the HRV signal. However, there is no standard approach for denoising HRV data, and current techniques are based on manually reviewing complete files, deleting abnormal beats, interpolating normal beats, and filtering the ECG^23^. Manual methods of noise removal can be very time-consuming with a high chance of human error. Deleting ectopic beats can lead to a systematic loss of information that can falsify the HRV analysis which is not ideal for clinical and epidemiological studies^24–26^. Presently, several approaches and algorithms are used including threshold filtering, wavelet transform methods, impulse rejection filter, three-dimensional spatial distribution mapping, automatic recursive filtering, interpolation methods, and predictive autocorrelation methods, among others for automated preprocessing with varied results^16,25^. Automated pre-processing has been included with several HRV analysis software packages including Kubios^27^, ARTiifact^28^, STREME^29^, and several open-source software written in MATLAB^30–32^.

The lack of a standard approach for the preprocessing of ECG signals before extracting the HRV indices, therefore, remains a major problem in HRV analysis. Traditionally, the preprocessing of ECG and HRV signals is considered separate steps. However, the existing denoising methods are only adaptable to one type of noise. In addition, most of these methods significantly distort the original signal removing useful information owing to a predetermined set of mathematical functions and projections. Some work on the effects of ectopic beats on HRV measures has been published previously^16,26,33–35^. While it is known that the majority of HRV measures are affected by ectopic beats there is a lack of quantitative analysis and in-depth studies. There have been no quantitative studies performed that compared the effects of both Gaussian noise and ectopic noise on HRV measures.

## Methods

### Data set

Forty, 75-min long artificial ECG files (ECG_art_) with a sampling frequency of 300 Hz were generated using the ECGSYN, a realistic ECG waveform generator in MATLAB^36,37^. These files are free of all noise and artifacts. QRS detector based on Pan Tompkins algorithm was used to obtain noise-free R-R intervals (RRI_art_)^38,39^. MATLAB was used for all the programming and statistical analysis in this study.

In addition, human ECG recordings from the following three Physionet^36^ databases were used: MIT-BIH Arrhythmia database^40^, MIT-BIH normal sinus rhythm database^41^, and the sudden cardiac death database^42^. The MIT BIH database recorded in the Arrhythmia Laboratory at Boston's Beth Israel Hospital (now the Beth Israel Deaconess Medical Centre) is the standard ECG database for analysis purposes. It includes the MIT BIH Arrhythmia database, which consists of 48 half-hour two-channel ECG records digitized at 360 Hz and 18 long-term ECG recordings at 128 Hz in the MIT BIH normal sinus rhythm database. The sudden cardiac death database consists of complete Holter recordings of 23 subjects with sudden cardiac death caused by ventricular fibrillation obtained from the Boston area hospitals. The open-source database is available at https://physionet.org/about/database/.

All 48 recordings of the MIT BIH Arrhythmia database were used in this study. The first 70 min of all the ECG recordings from the MIT BIH normal sinus rhythm and sudden cardiac death database were extracted for analysis. The RRIs were derived by applying the QRS detector based on Pan Tompkins algorithm in MATLAB.

### Addition of Gaussian noise and ectopic beats

Programs written in MATLAB were used to add Gaussian and ectopic noise in the ECG_art_ files. White Gaussian noise was added to each of the 40 ECG_art_ files at intervals of 2 dB, 4 dB, 6 dB, 8 dB, and 10 dB of noise. The Gaussian noise was generated by the MATLAB code *awgn.m*. These ECG_art_ with embedded Gaussian noise (ECG_gau)_ were free from all other artifacts. RR intervals with Gaussian noise (RRI_gau_) were then derived by the Pan Tompkins algorithm.

A single QRS complex was extracted from the ECG_art_ signal to model the ectopic beats as an additional heartbeat in the ECG signal. A specified percentage of ectopic beats at 2%, 4%, 6%,8%, and 10% was added to each of the ECG_art_ files to generate ECG with ectopic noise (ECG_ect_). The location of the artifacts in the time series was chosen randomly based on the position of the R peaks in the ECG_art_ signal so that the added beat does not overlap with an existing beat. The amplitude of the R peak of QRS complex for each ECG_art_ file was adjusted by a factor of the mean value of the amplitude of the R peaks of ECG_art_ over the amplitude of the R peak of QRS complex.

The Pan Tompkins was modified to identify the additional ectopic beats which would not be detected otherwise, by lowering the fiducial threshold. RR intervals with ectopic noise (RRI_ect_) were then calculated. The length of the signal was preserved by removing the RR intervals adjacent to the added artifact in the RRI_ect_.

### Pre-processing tool for Gaussian noise and ectopic beats

#### Complete ensemble empirical mode decomposition with adaptive noise (CEEEMDAN)

Empirical mode decomposition (EMD) is a local adaptive algorithm that decomposes non-stationary multi-component signals to amplitude and frequency modulated (AM–FM) intrinsic mode functions (IMF). The overall result is a separation of the signal into ordered modes ranging from high frequency to low frequency in the time domain following the process below^43^:

1. Identify all local extrema in the signal, *x*(*t*)*.*
2. Create the upper and the lower envelop by connecting all the maxima and minima through cubic spline.
3. Compute the mean function, local mean, of upper and lower envelop, *m*(*t*)
4. Calculate the difference, *d*(*t*) = *x*(*t*) − *m*(*t*)*.*
5. For *d*(*t*), to be the first IMF, *c*_1_(*t*), it must satisfy two conditions:
  - The number of extrema and zero-crossing must not differ by more than one.
  - The local mean *m*(*t*) must be close to zero at any given point.
6. The residual signal is calculated as *r*(*t*) = *d*(*t*)* − c*_1_(*t*).
7. Repeat steps (1) to (6) to obtain more IMFs until predefined stopping criteria are met or when the final residual signal is obtained as a monotonic signal.

However, the EMD algorithm presents a major problem of mode mixing as each mode is preferred to have similar scales of frequency. This phenomenon presents as variable scales of oscillations in one mode or similar oscillations in different modes. Several new methods and improvements have been proposed to address the mode mixing issue of EMD algorithm. One of these approaches is the ensemble empirical mode decomposition (EEMD). EEMD adds white Gaussian noise to the signal and performs decomposition of the original signal over an ensemble of added noise^44^. The EEMD follows the same algorithm as the EMD, and the final modes are calculated by averaging the corresponding IMFs in each iteration. Even though EEMD reduces mode mixing seen in EMD, several modes from different realizations of the signal and noise are still produced. In addition, residual noise can be found in the reconstructed signal. Complete ensemble empirical mode decomposition with adaptive noise (CEEMDAN) is then a further improvement on EEMD which resolves these issues^45^.

#### Single dependent rank order mean (SDROM)

A single dependent rank order mean (SDROM) algorithm was used to filter impulse noise from image^46^ and sound^47^. The algorithm identifies the corrupted values by comparing them with neighboring samples and works by using a 1D odd sliding window of size n to look at a sample segment X centered at X(n). Z(n) is the vector of size n − 1 that excludes the sample of inspection, X(n). Z(n) is then sorted in an ascending order, R(n) and the rank ordered mean (ROM), $\mu$ $\mu = \frac{{R}_{\frac{n-1 }{2}}\left(n\right)+ {R}_{\frac{n-1 }{2}+1}(n)}{2}$ based on the window size. The impulse noise threshold T_i_ is chosen such that $i=\frac{n-1}{2}$ . The rank-ordered differences D_i_(n) are then calculated as

1 $${D}_{i}\left(n\right)=\left\{\begin{array}{c}{R}_{i}\left(n\right)-x\left(n\right) , x\left(n\right)\le \mu \\ X\left(n\right) - {R}_{\left(n-1-i\right)}\left(n\right), x\left(n\right)>\mu \end{array}\right.$$

where X(n) is noise if ${D}_{i}\left(n\right)>{T}_{i}$ conditions are met^47^.

#### Adaptive filtering algorithm (ADF)

The adaptive filtering algorithm (ADF) is based on the adaptive mean value and standard deviation values which change and adjust in accordance with the variability of the time series. A binominal filtered series, T(n) estimating the heart rate variability is calculated using the tachogram or RRI. This filtered series mimics the HRV without any artifacts. The adaptive mean and the adaptive standard deviation of this series are calculated, and a controlling coefficient regulates the adaptive mean. An individual RRI is classified as abnormal if it fails to meet the thresholds defined by the proportional limit and adaptive standard deviation. An abnormal RRI is replaced by values calculated from the adaptive mean and standard deviation. The next step is the final adaptive controlling procedure where the filtered series is rechecked based on a filter coefficient and a basic variability parameter which reduces filtering errors by maintaining the variability of the RR intervals^48^. ADF is a dynamic and self-correcting filter that spontaneously adapts the filter coefficients to the time series to preserve the variability and characteristics of the data.

### Proposed approach

The CEEMDAN code written in MATLAB by Colominas and collaborators was used to remove the Gaussian noise from the ECG_gau_ files after dividing the signal into multiple segments of shorter length to decrease computational cost^44,45^. The code outputs the different modes ordered by frequency of the ECG_gau_ signal separating the Gaussian noise and the ECG signal.

CEEMDAN was then applied to each segment separately with noise standard deviation (Nstd) of 0.2, 500 realizations (NR), and maximum iterations of 5000 (MaxIter)^49^. The next step was to apply the fast Fourier transform to each mode to define its frequency domain. Then the mean frequency (*f*_*m*_) of each mode was calculated by averaging the frequency values for which the amplitude was greater than one-fourth of the maximum amplitude for that mode respectively. If *f*_m_ was greater than the noise threshold frequency of 35 Hz the mode was classified as noise and removed. The ECG segment was then reconstructed using the remaining modes. It has been reported that for clean and noisy ECG signals the first three modes contain the noise and the QRS complex^50^. The noise threshold set based on the power spectrum of each mode was not very high and to remove the remaining noise in the reconstructed ECG segment a hybrid approach was used. A level one wavelet decomposition with soft thresholding was applied to the reconstructed segment of the denoised ECG to remove the noise in the remaining IMFs. After each segment was processed through the filter, the segments were concatenated to form the reconstructed denoised ECG signal.

A comparison study for removing an increasing percentage of ectopic noise from RRI_ect_ by the SDROM, ADF, and the two combinations of these filters: SDROM-ADF and ADF-SDROM was then conducted. Forty RRI_ect_ files corrupted with an increasing percentage of ectopic beats were filtered using these four methods. The Pearson correlation coefficient, *ρ*, and root mean square error (RMSE) were used as performance metrics for all the filters used. *ρ* is described as*:*

2 $$\rho =\frac{1}{N-1 }\sum_{i=1}^{N}\left(\frac{xi-\mu x}{\sigma x}\right)\left(\frac{yi-\mu y}{\sigma y}\right)$$

and RMSE is defined as:

3 $$RMSE=\sqrt{\frac{\sum_{i=1}^{N}{\left(xi-yi\right)}^{2}}{N}}$$

where $xi$ is the original noise-free signal and $yi$ is the denoised signal.

### Statistical analysis

Twenty-four of the most common HRV features from the time domain, frequency domain, non-linear measures, and fragmentation measures were calculated for the artificial and real signals ^51^. A paired *t*-test was used to calculate the statistical difference between HRV measures with and without noise for artificial RRI. Another paired *t*-test was conducted between the noisy and the denoised HRV measures for real signals. In addition, for the artificial signals, the relative percentage change in HRV features (RHRV) was calculated as a measure of change relative to the percentage of added ectopic beats. RHRV was calculated as:

4 $$RHRV= \frac{{HRV}_{ect}-{HRV}_{ref}}{{HRV}_{ref}} \times 100$$

where ${HRV}_{ect}$ is the HRV result with a certain percentage of added ectopic beats and ${HRV}_{ref}$ is the original noise-free HRV result. A linear regression analysis was performed, and the results were ranked by the absolute value of the slope of the regression line (B value) for each group (time domain, frequency domain, nonlinear measures, and fragmentation measures).

## Results

In this section, the results obtained from the denoising of artificial and real noisy ECG signals using the proposed approach will be discussed. In addition, the effects of different levels of Gaussian and ectopic noise on HRV features will be analyzed.

### Validation of the pre-processing tool

**Figure 1:**
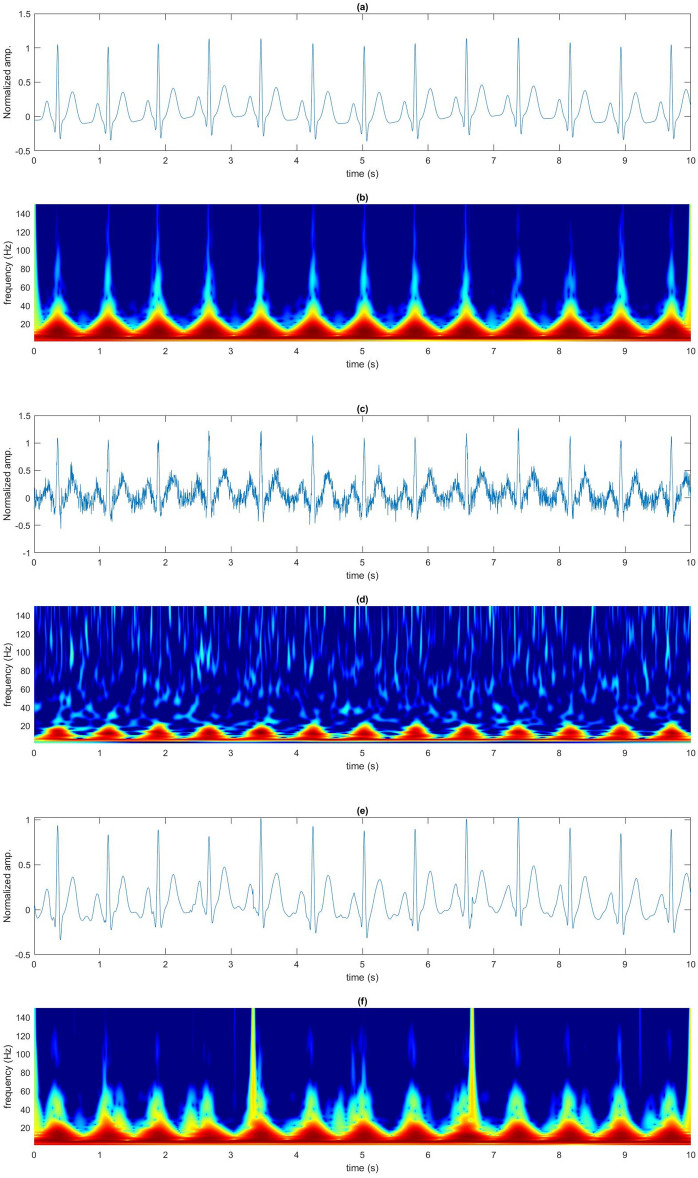
Time-frequecy domain for CEEMDAN-WD denoising (**a**) Waveform of original artificial ECG signal, (**b**) Spectrogram of original artificial ECG, (**c**) Waveform of artificial ECG signal with 2 dB of white Gaussian noise, (**d**) Spectrogram of artificial ECG signal with 2 dB of white Gaussian noise, (**e**) Waveform of partially reconstructed denoised ECG signal by CEEMDAN-WD proposed method, (**f**) Waveform of partially reconstructed denoised ECG signal by CEEMDAN-WD proposed method.

Figure 1 shows the performance of the proposed approach used to denoise ECG_gau_ with 2 dB of added Gaussian noise. The denoising result in the time–frequency domain shows high visual quality and accuracy of the partially reconstructed denoised ECG (ECG_den_) with a significant noise reduction. The spectrograms in Fig. 1 demonstrate that the energy profile of the reconstructed ECG signal, specifically of the QRS complex is preserved after denoising with the CEEMDAN-WD method.

**Figure 2:**
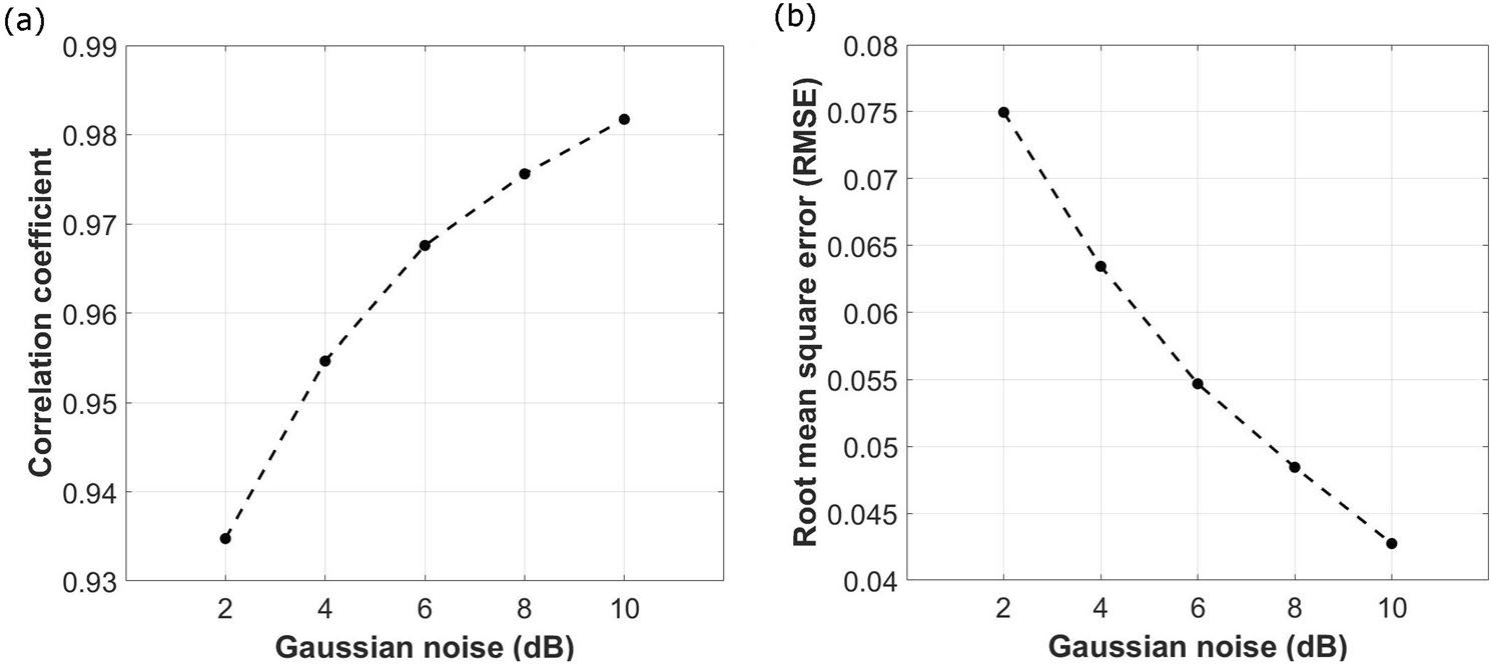
Performance indicators for CEEMDAN-WD (**a**), Mean correlation coefficient for CEEMDAN-WD denoising algorithm at different levels of Gaussian noise, (**b**)**,** Mean RMSE for CEEMDAN-WD denoising algorithm at different levels of Gaussian noise.

The performance indicators for denoising the ECG_gau_ with the proposed method are presented in Fig. 2. High mean correlation coefficient values indicate good reconstruction of the ECG_den_ in comparison to the original ECG. The correlation coefficient value increase with an increase in the SNR level of Gaussian noise (decreased noise). The accuracy of the designed filter is further reaffirmed by the small RMSE values seen in Fig. 2b. The trendline shows a decrease in the RMSE values with a decrease in the level of Gaussian noise (high SNR level) indicating overall good performance for the proposed approach.

A comparative analysis in terms of the RMSE of the CEEMDAN-WD filtering and some EMD domain denoising techniques using real ECG signals was performed. Three ECG recordings from the MIT- BIH Arrhythmia database, ‘111’,’116’, and ‘205’, were embedded with 10 dB Gaussian noise and filtered using the CEEMDAN-WD. The RMSE values for the EMD and EEMD based direct subtraction (EMD-DS, EEMD-DS)^52^, EMD with Kullback Leibler Divergence (EMD-KLD)^53^, and CEEMDAN with interval thresholding and higher order statistics (CEEMDAN-HIT) for the same ECG recordings were taken from literature^54^. The performance for each method is shown in Table 1. As seen from this table, lower RMSE values are given by the CEEMDAN-WD method.

**Table 1:** RMSE performance values for comparative analysis of the proposed CEEMDAN-WD method versus some developed methods EMD-DS, EEMD-DS, EMD-KLD and CEEMDAN-HT.

ECG recording	EMD-DS	EEMD-DS	EMD-KLD	CEEMDAN-HIT	CEEMDAN-WD
111	7.02	8.38	14.26	4.94	0.0439
116	11.86	10.91	11.86	8.55	0.1652
205	8.19	13.87	8.19	4.96	0.0732

**Figure 3:**
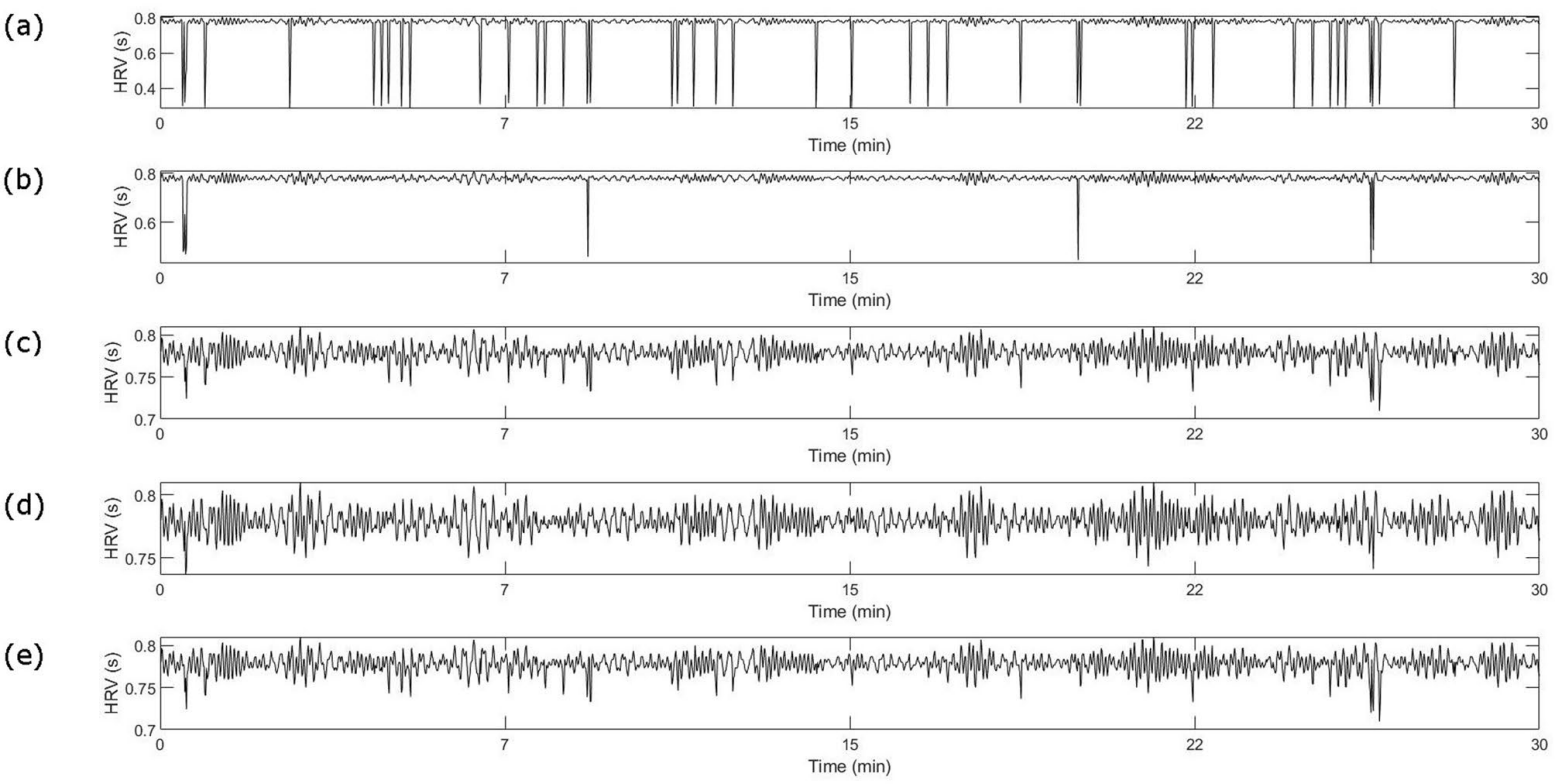
Comparison of different ectopic filters, (**a**) Artificial RR interval with 2% of ectopic beats, (**b**) Denoised RR interval by SDROM, (**c**) Denoised RR interval by ADF, (**d**) Denoised RR interval by combination filter SDROM-ADF, (**e**) Denoised RR interval by combination filter ADF-SDROM.

The second type of noise, ectopic beats, are processed through two different filtering algorithms and their combinations to evaluate the robustness and performance of each approach. Figure 3 illustrates the denoising results of ECG_ect_ with 2% of added ectopic noise for each approach. As seen clearly in the figure, the filter combinations, SDROM-ADF and ADF-SDROM, outperform the individual algorithms which fail to account for all the ectopic beats in the signal. However, between the two filter combinations, SDROM-ADF shows a better visual performance in removing the ectopic noise.

**Figure 4:**
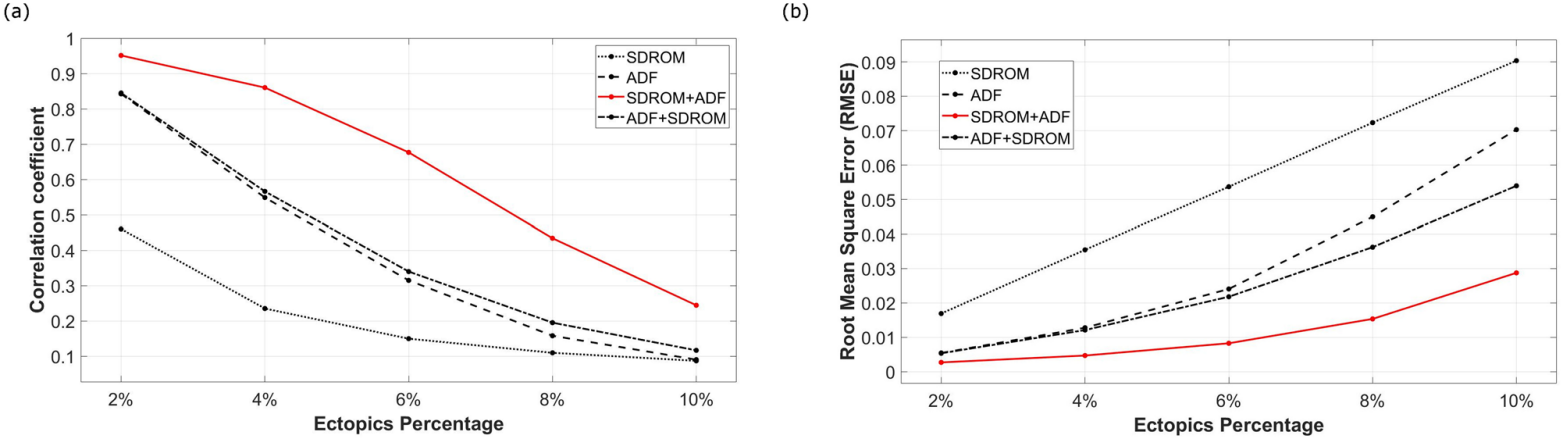
Comparison of performance indicators for ectopic filters (**a**)**,** Mean correlation coefficient for SDROM, ADF, SDROM-ADF, and ADF-SDROM denoising algorithm at different levels of ectopic noise, (**b**), Mean RMSE for SDROM, ADF, SDROM-ADF, and ADF-SDROM denoising algorithm at different levels of ectopic noise.

The performance of each approach is quantified by the mean correlation coefficient and RMSE values. Figure 4 shows the correlation coefficient and RMSE values for all four methods.

SDROM has the lowest correlation coefficients for all noise levels whereas the ADF filter and the combination filter ADF-SDROM show similar results at low levels of ectopic noise. However, ADF- SDROM filter performs better than the ADF filter at higher levels of noise as seen in Fig. 4a. SDROM-ADF method has the highest correlation coefficient values for all levels of noise and shows the best performance.

Figure 4b illustrates the RMSE values for all four filters. SDROM- ADF filter has the lowest values for all levels of noise and SDROM filter has the highest values of RMSE for all percentages of added ectopic beats. ADF and ADF-SDROM filters have similar performance for lower levels of noise with ADF- SDROM performing better at a higher percentage of added ectopic beats.

Therefore, a two-step denoising method was applied where the first step is to denoise the ECG signal using the CEEMDAN-WD algorithm to remove Gaussian noise followed by the application of the SDROM- ADF filter to remove ectopic noise. The complete denoising algorithm was applied to 40 artificial ECG signals with embedded Gaussian noise of 6 dB and 6% of added ectopic beats. The proposed method showed good performance with a mean correlation coefficient of 0.846 ± 0.114 (mean ± SD) and an RMSE value of 7.69 × 10^–5^ ± 4.86 × 10^–4^.

**Figure 5:**
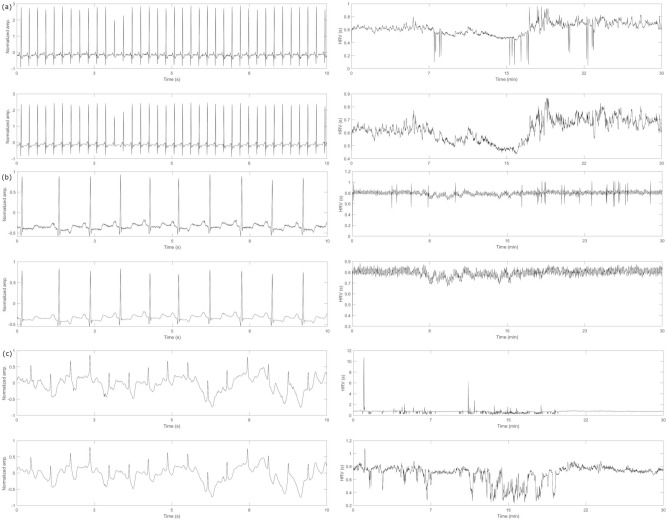
Proposed method applied to real ECG signals, (**a**) Denoising of the ‘16,539’ ECG recording from the MIT-BIH normal sinus rhythm database, CEEMDAN-WD filtering Gaussian noise (left), and SDROM-ADF filtering ectopic beats (right), (**b**) Denoising of the ‘119’ ECG recording from the MIT-BIH Arrhythmia database, CEEMDAN-WD filtering Gaussian noise (left) and SDROM-ADF filtering ectopic beats (right), (**c**) Denoising of the ‘52’ ECG recording from the sudden cardiac death database, CEEMDAN-WD filtering Gaussian noise (left) and SDROM-ADF filtering ectopic beats (right).

Figure 5 shows a visual illustration of the denoising approach applied to one ECG signal from each of the real ECG signal databases selected above. Figure 5a–c show the denoising of the ‘16,539’, ‘119’, and ‘52’ ECG recording from the MIT-BIH normal sinus rhythm, the MIT-BIH Arrhythmia, and the sudden cardiac death database respectively. It can be clearly seen from these figures that the denoising performance on the real ECG signals is showing similar impressive results. The CEEMDAN-WD successfully removes the Gaussian noise without altering the original ECG signal and the SDROM-ADF approach removes most of the ectopic noise seen in the HRV.

### Effects of noise type on HRV measures

Twenty-four HRV features were extracted from the RRI_art,_ RRI_gau_ and RRI_ect_ files. Time-domain features which define the variability of beat to beat intervals include the average N-to-N intervals (AVNN), the standard deviation of the N-to-N intervals (SDNN), the root mean square of differences between successive N-to-N intervals, the percentage of N-to-N intervals greater than 50 ms (pNN50) and standard error of the mean N-to-N interval (SEM). Frequency-domain features measure the distribution of power into the following discrete frequency bands: high-frequency band (HF, 0.15–0.4 Hz), low-frequency (LF, 0.04–0.15 Hz), and very low-frequency (VLF, 0.0033– 0.04 Hz). The frequency-domain features calculated were total power (combine power in all three bands), VLF power, LF power, HF power, normalized VLF power (VLF Norm), normalized LF power (LF Norm), normalized HF power(HF Norm), the ratio between LF and HF power (LF to HF), LF peak and HF peak^4,23^. Nonlinear measures reflect the complexity and unpredictability of the HRV signal. These measures included SD1 (standard deviation of N-to-N intervals along the perpendicular to the line of identity), SD2 (standard deviation of N-to-N intervals along the line of identity), alpha1 (low scale slope of detrended fluctuation analysis), alpha2 (high scale slope of detrended fluctuation analysis) and sample entropy^55,56^. Fragmentation features include PIP (percentage of inflection points in the N-to-N interval), IALS ( Inverse average length of segments), PSS (percentage of short segments), PAS (percentage alternation segments)^57^.

**Figure 6:**
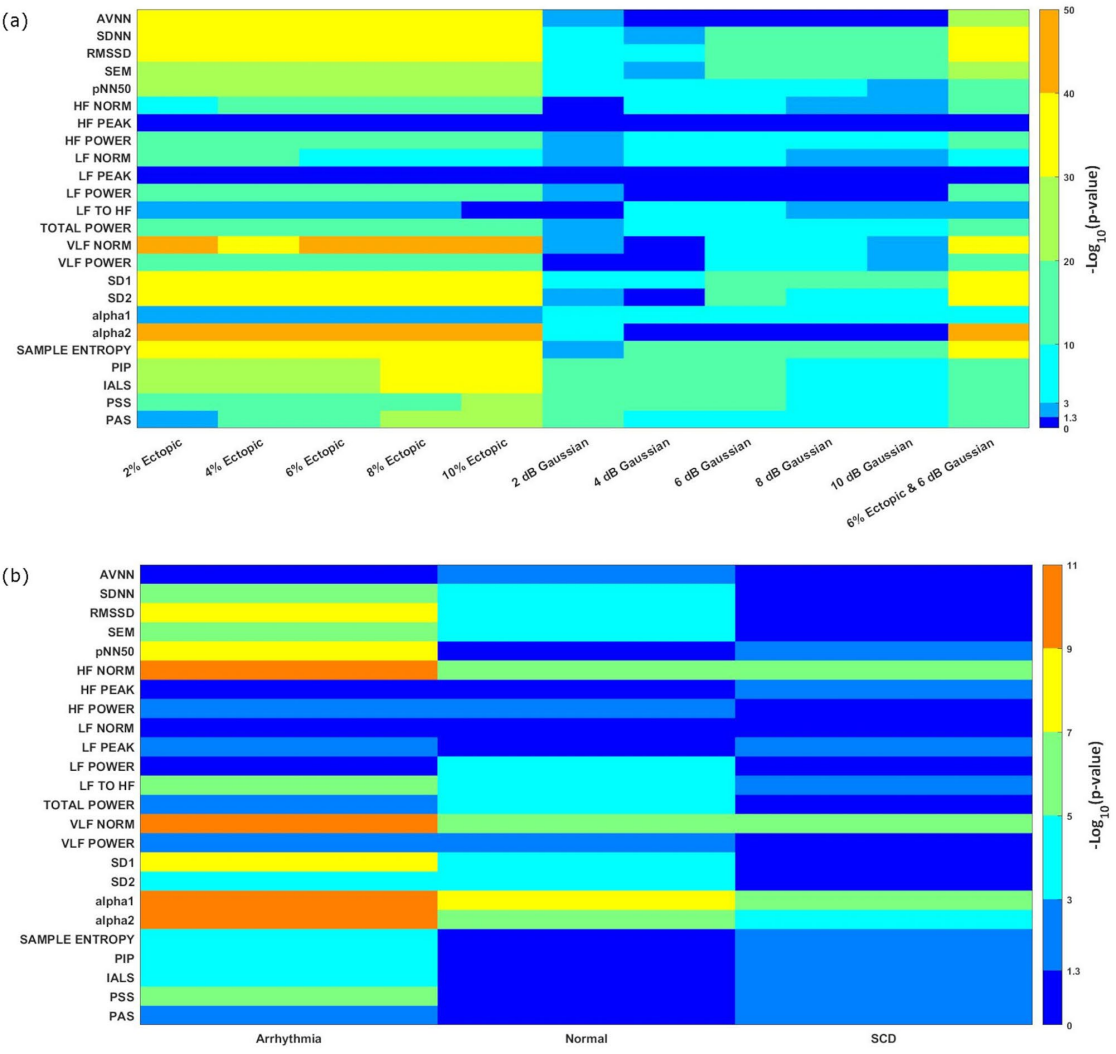
Heat maps for −log_10_ transformed p-values for twenty-four of the most common HRV measures for real and artificial signals, (**a**) Heat map of −log_10_(p-value) of original clean and noisy artificial HRV measures for different levels of ectopic and Gaussian noise. The last column shows the artificial HRV measures for both types of noise combined at 6 dB of Gaussian and 6% of ectopic noise, (**b**) Heat map of −log_10_(p-value) of noisy and denoised (by the proposed algorithm) real HRV measures obtained from three Physionet databases: MIT-BIH Arrhythmia, MIT-BIH Normal Sinus Rhythm, and Sudden Cardiac Death database.

The results of the paired t-test are shown in Fig. 6. *p* values with large variability cannot provide support as accurate and reliable measures of evidence against the null hypothesis^58^. One way to resolve this issue is to explicate the *p-value* expressed as *p* = *c* × *10*^*-k*^ on a log scale as −log_10_(*p*) = −log_10_(*c*) + *k* where *c* is a constant and *k* an integer. This implies that the magnitude k is a continuous measure of the actual strength of evidence^58,59^. Using the −log_10_(*p*) value significant statistical differences between the HRV of clean and noisy signals for each HRV measure for different levels of noise were able to be ranked.

The comparison of original and noisy artificial HRV measures is shown in Fig. 6a. All the HRV measures apart from HF peak and LF peak (−log_10_(*p*) < *1.3*) were statistically significant for ectopic noise. VLF norm (except for 4% ectopic noise) and alpha2 resulted in the highest *k* value followed by AVNN, SDNN, RMSSD, SD1, SD2, and sample entropy. Whereas LF to HF, alpha 1, and PAS for 2% of added ectopic beats resulted in the least order of difference.

AVNN (except 2 dB of Gaussian noise), HF peak, LF peak, LF power, and alpha 2(except 2 dB of Gaussian noise) were not significantly affected by the addition of Gaussian noise. The highest order of significant change was observed for the fragmentation measures for higher levels of Gaussian noise. In addition, some HRV measures such as SDNN, RMSSD, SEM, and non-linear measures: SD1, and sample entropy showed the most significant changes for lower levels of Gaussian noise.

The last grid in Fig. 6a shows the change for HRV measures with both Gaussian and ectopic noise as would be the case with real signals, indicating a significant change in all the HRV measures except HF peak and LF peak, which were not affected by either type of noise.

Figure 6b illustrates the findings for the paired t-test between the noisy and denoised real HRV measures. The results reaffirm that most HRV measures are sensitive to noise and denoising the signal before performing HRV analysis will alter the outcome of the results. The HRV measures obtained from the MIT-BIH Arrhythmia database showed no significant change for AVNN, HF peak, LF norm, and LF power between the noisy and denoised signals. HF norm, VLF norm, alpha1, and alpha 2 show the highest statistical significance. The MIT BIH-normal sinus rhythm database showed no effect on the fragmentation measures, sample entropy, HF peak, LF norm, LF peak, and pNN50. The biggest change for this database was in the nonlinear measure, alpha1 followed by alpha2, VLF norm, and HF norm. The most significant difference for the sudden cardiac death database was HF norm, VLF norm, and alpha1 whereas most of the time and frequency domain measures, as well as SD1 and SD2, did not result in any significant change.

**Figure 7:**
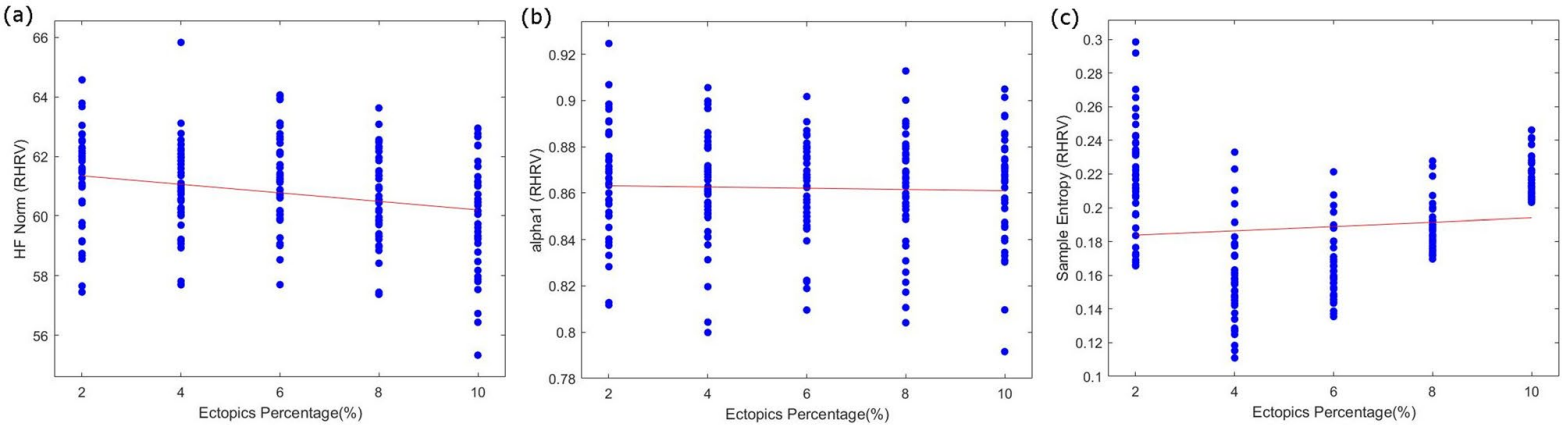
Examples of HRV measures that did not show a linear relationship in the relative change of HRV measure (y axis) with an increasing percentage of ectopic noise (x axis), (**a**) HF-Norm, (**b**) alpha 1, (**c**) Sample entropy.

Table 2 shows the results of the linear regression analysis ranked by the absolute value of the slope (*B* value). The majority of the HRV features examined for added ectopic noise indicate a linear relationship between the relative percentage change in HRV features (RHRV) and the percentage of added ectopic noise. Some HRV measures such as HF norm, alpha1, and sample entropy among others do not show a linear change with an increasing percentage of artifacts. RHRV for these measures when plotted against the increasing percentage of artifacts showed a distinct widening of the spread of data points with small slope values as shown in Fig. 7. Subsequently, the *p* values for the regression model of these measures were not significant.

**Table 2:** HRV measures ranked by absolute value of slope (B) on linear regression grouped by time domain measures, frequency domain measures and non-linear and fragmentation measures.

Ectopic noise					Gaussian noise				
HRV Measures	R^2^	*p*	B	Abs(B)	HRV measures	R^2^	*p*	B	Abs(B)
SEM	0.889	< 0.0001	0.016	0.016	SEM	0.111	< 0.0001	−4.41E−04	4.41E−04
pNN50	0.557	< 0.0001	0.646	0.646	pNN50	0.088	< 0.0001	0.002	0.002
AVNN	0.999	< 0.0001	−0.930	0.930	SDNN	0.112	< 0.0001	−0.034	0.034
SDNN	0.970	< 0.0001	1.225	1.225	RMSSD	0.136	< 0.0001	−0.046	0.046
RMSSD	0.948	< 0.0001	1.355	1.355	pNN50	0.208	< 0.0001	−0.270	0.270
LF PEAK	0.000	**0.943**	−0.006	0.006	HF PEAK	0.011	**0.133**	0.005	0.005
HF PEAK	0.013	**0.104**	0.342	0.342	LF PEAK	0.002	**0.487**	0.005	0.005
LF TO HF	0.010	**0.155**	0.403	0.403	LF TO HF	0.000	**0.919**	0.011	0.011
VLF NORM	0.010	**0.155**	7.208	7.208	LF NORM	0.096	< 0.0001	15.287	15.287
LF NORM	0.007	**0.249**	13.873	13.873	HF NORM	0.032	0.011	21.845	21.845
HF NORM	0.014	**0.096**	−21.098	21.098	VLF NORM	0.061	4.02E−04	−35.935	35.935
VLF POWER	0.611	< 0.0001	17,416.08	17,416.08	VLF POWER	0.030	0.014	−91.591	91.591
LF POWER	0.644	< 0.0001	51,760.12	51,760.12	LF POWER	0.057	0.001	−138.717	138.717
HF POWER	0.622	< 0.0001	102,963.27	102,963.27	HF POWER	0.071	1.42E−04	−245.000	245.000
TOTAL POWER	0.636	< 0.0001	172,579.35	172,579.35	TOTAL POWER	0.060	4.84E−04	−477.852	477.852
alpha1	4.969E−05	**0.921**	−0.026	0.026	SD1	0.136	< 0.0001	−0.033	0.033
SAMPLE ENTROPY	0.004	**0.346**	0.210	0.210	SD2	0.102	< 0.0001	−0.037	0.037
alpha2	0.014	**0.092**	0.297	0.297	alpha1	0.130	< 0.0001	0.603	0.603
SD1	0.948	< 0.0001	0.958	0.958	IALS	0.309	< 0.0001	−0.903	0.903
IALS	0.751	< 0.0001	1.307	1.307	SAMPLE ENTROPY	0.050	0.002	−1.681	1.681
SD2	0.969	< 0.0001	1.443	1.443	alpha2	0.107	< 0.0001	−1.774	1.774
PAS	0.826	< 0.0001	51.035	51.035	PAS	0.323	< 0.0001	−47.178	47.178
PIP	0.751	< 0.0001	130.640	130.640	PIP	0.309	< 0.0001	−90.261	90.261
PSS	0.575	< 0.0001	199.337	199.337	PSS	0.329	< 0.0001	−106.038	106.038

Non-significant values are bold.

Time-domain and frequency-domain measures except for AVNN, LF peak, and HF norm showed increasing variability with the increase in the percentage of added ectopic beats. Among the time domain measures, SDNN and RMSSD were more sensitive to added artifacts than other measures. However, SEM was robust to ectopic noise. No clear distinction between short-term and long-term time-domain metrics was observed. In addition, absolute power measures from the frequency domain were more sensitive to added artifacts when compared to normalized power metrics.

All non-linear and fragmentation measures except alpha1 increased in HRV with the increase in added artifacts. The non-linear measures SD2 and SD1 were the most sensitive to ectopic noise whereas all fragmentation measures showed the greatest sensitivity to ectopic noise.

**Figure 8:**
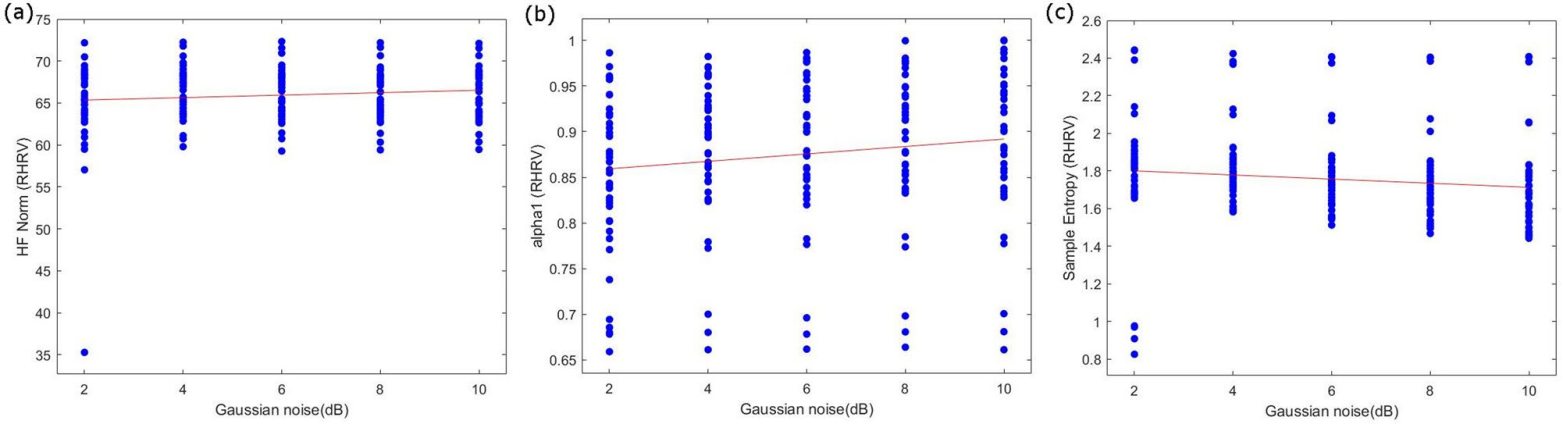
Examples of HRV measures that did not show a linear relationship in the relative change of HRV measure (y axis) with incrementing Gaussian noise (x axis), (**a**) HF-Norm, (**b**) alpha 1, (**c**) Sample entropy.

For the majority of HRV measures, the variability in RHRV with increasing SNR of Gaussian noise was comparatively large. This results in an increased spread of data, especially for lower SNR values, like what was observed for some HRV measures for ectopic noise. These measures do not conform well to straight line fitting despite having a highly significant *p-value* as displayed in Fig. 8.

## Discussion

Holter recordings and the latest developments in computational tools have enabled HRV analysis to be used in clinical settings. HRV analysis is proving to be crucial for risk stratification and prognosis of various cardiovascular conditions as well as chronic disease classification and progression. In addition, changes in HRV in unrelated diseases such as stroke^60^, dementia^61^, mental illness^62^, renal failure, diabetes, sleep apnea, stress, and pain among others have been observed^5,63,64^. Noise in the recording, being technical or ectopic beats influence any analysis of the variance in the signal. It is thus imperative to remove all artifacts from the ECG signal and RRI and consider the sensitivity of the HRV measures to Gaussian and ectopic noise before performing HRV analysis to avoid any false conclusions. A robust automated pre-processing tool based on a two-step hybrid filtering approach for HRV analysis is proposed in this study to address some of the shortcomings in the current methods applied for preprocessing of ECGs or RRIs.

Technical noise in the ECG can distort the morphology of the signal and make accurate detection of the R peak difficult. There are several QRS detecting algorithms and their mechanisms can be generally divided into two stages, the preprocessing, and the decision-making stages. The preprocessing stage usually consists of linear filtering and nonlinear transformations where the ECG waveform is smoothed and amplified. The decision-making stage then classifies the QRS and non-QRS waves^65^. A comparison study of 10 QRS detectors including the Pan Tompkins and Hamilton’s shows that most QRS detectors regardless of the mechanism have similar sensitivity, positive predictivity, and detection accuracy with noise-free or high-quality ECG signals whereas detection accuracy significantly decreases with low-quality and noisy ECG^66^. A review of QRS detecting algorithms on the MIT BIH Arrhythmia database corroborates these findings^65^. Most studies conducted on ECG noise analysis and QRS detection algorithms used cleaned data for evaluation and overall performance without providing details of specific noise types and intensity levels of technical noise that can affect the QRS morphology nor the preprocessing steps and algorithms applied. This makes an accurate comparison between heartbeat detectors mentioned in the literature difficult.

One study compared the performance of the Pan Tompkins, Hamilton, and WQRS (Physionet) algorithms for heartbeat detection on clean and noisy simulated ECG with baseline wander, muscle artifact, and electrode motion artifacts^67^. The algorithms performed well on high-quality signals, but performance decreased for signals contaminated with noise. The Pan Tompkins algorithm had the best performance when dealing with noisy signals. The experimental results on the characteristics and intensity of artifacts and beat detection indicated that electrode motion and muscle artifacts, modeled as Gaussian noise in the present study, have the most influence on the detection of QRS leading to a high number of misdetection and false detections. Baseline wander noise has a lesser effect on the detection algorithms and is usually removed by the bandpass or lowpass filters applied in most detectors^65,67^. Therefore, removing technical noise from the ECG signal before applying a heartbeat detection algorithm can greatly improve the detection performance and accuracy of these algorithms. Time–frequency analysis and sub-band decomposition algorithms based on linear, adaptive and Baysian filtering combined with signal quality index have shown relative success in removing high frequency noise and motion artifacts from ECG signals^68,69^. These techniques are also applied in conjunction with machine learning algorithms to classify abnormal waveforms or signal^68,70^.

Traditional ECG denoising methods such as wavelet decomposition assume prior information of the signal and type of noise, which is hard to obtain in clinical settings. Many linear approaches to filtering are not stable for non-linear and non-stationary ECG signals^71^. EMD-based denoising is an adaptive data-driven approach and hence has shown superior performance to other approaches. A comparative performance analysis of discrete wavelet transform (DWT), EMD, and EEMD showed that EMD-based methods outperformed DWT^72,73^. In another study, EMD and EEMD-based denoising of ECG showed better results than the Wiener filter to remove Gaussian noise^52^.

The basic principle of CEEMDAN-based denoising applied here is a partial reconstruction of the ECG signal after decomposition to its IMFs by removing irrelevant low-order modes identified by a frequency threshold that is based on a fast Fourier transform. To identify the relevant and irrelevant modes for partial reconstruction of the denoised ECG direct subtraction methods of lower order IMFs^52^, the energy density of IMFs^74^, moving average filter^50^, probability density function similarity measure based on Kullback Leibler Divergence between input signal and IMFs ^53^and detrended fluctuation analysis threshold^75^ have been used. Other methods applied thresholding techniques to all, or some IMFs based on the quantity of noise to retain the QRS complex in lower order IMFs that are excluded in the partial reconstruction of the ECG signal. These methods included wavelet thresholding^76^, moving average filter^50^and interval thresholding, and higher order statistics^54^. In this study, a hybrid approach using wavelet transform on the partially reconstructed ECG signal from CEEMDAN was preferred to improve the denoising method.

Moreover, a comparative analysis of the proposed technique with some of the recently developed EMD-based denoising techniques confirms the superiority of the proposed CEEMDAN-WD method. The literature reports in agreement with the observations in this study that CEMDAN is more accurate and suitable for denoising ECG applications when compared to EMD and EEMD methods^45,54,71,77–81^. It is important to note that the CEEMDAN-based methods have the lowest RMSE values. EMD-DS and EMD-KLD methods show similar performance for some ECG records. This might be because both techniques discard the first IMF to reconstruct the denoised ECG signal. It was, therefore, surprising that the EEMD-DS underperformed the EMD methods for one of the ECG records.

Research shows that all editing methods affect HRV analysis. The degree of impact depends on the editing method, length of the signal, and percentage of ectopic noise^25^. Therefore, it is imperative to choose appropriate editing methods without influencing the results. This study applied a spontaneous adaptive filtering approach that can self-adjust to the peculiarities of each individual signal keeping the variability of the HRV analysis intact. ADF-based filtering has been used in different HRV studies with good results^82,83^. Applying an impulse noise filter such as SD-ROM before ADF significantly improved the performance of the filter. This proposed approach has shown good performance in previous work^84^.

The presence of even a small number of ectopic beats can significantly alter HRV analysis results^16,24,26,33,34^. One study showed that even less than 1% of ectopic beats in the HRV signal were enough to cause a significant difference in the analysis^34^. Zhao et al. demonstrated the effect of ectopic beats on HRV analysis increases with the increase in the number of ectopic beats^33^. Researchers have also found that the presence of ectopic beats can change the HRV analysis results for congestive heart failure patients^85^. Our analysis of noise-free and noisy artificial signals confirmed these results. Both types of noise significantly affect the majority of the HRV measures. A greater number of HRV measures seem to be affected by ectopic noise than Gaussian noise from our analysis using −log_10_(*p*). It is important to keep in mind that −log_10_(*p*) cannot quantify the change caused in the HRV measures by the increasing percentage of noise. It merely represents the strength of evidence that confirms the change.

There has been some work published on the analysis of ectopic beats on the time domain, frequency domain, and non-linear measures^16^. However, these studies do not consider the changes in the fragmentation indices, which are included in our study. Time domain measures calculated from successive differences have been reported to be more sensitive to noise and measures that represent the variation such as AVNN and SDNN are more resilient to noise^16^. On the contrary, our results from Table 2 show that AVNN and SDNN are more sensitive to ectopic noise with RMSSD being the most sensitive. While all the time domain measures studied show an increase with an increasing percentage of ectopic beats, AVNN decreases with increasing ectopic noise. Overall, the time domain measures are less sensitive to Gaussian noise with pNN50 being the most affected.

It has been found that only one ectopic beat in a 2-min-long ECG recording can lead to a 10% increase in HF power^16^. This change in all the frequency bands has been found in the current results. There is an increase in the power bands of the frequency domain with the increase in ectopic noise. The only exception to this is the normalized HF power, which demonstrated a decrease with an increase in the ectopic noise. The sensitivity of frequency indices is based on the method of calculation. The relative power measures are more sensitive to ectopic noise than normalized power measures. However, it is important to note that the linear regression model calculated for the normalized power measures does not fit well due to the widespread data as seen in Fig. 7. Similar results have been observed for the Gaussian noise. Frequency domain measures appear to be the most sensitive to both ectopic and Gaussian noise.

A decrease in the values of sample entropy with the presence of ectopic beats has been reported. This is in agreement with the results observed from the calculation of RHRV from the current signals. At 2% ectopic presence in the HRV a decrease of approximately 198% was observed. Similarly, percentage changes in detrended fluctuation analysis have also been observed. But these changes do not significantly increase with the increase of ectopic noise as is seen in Fig. 7. The Poincaré plot measures however are significantly affected by ectopic noise with SD2 showing increasing variability with increasing noise leading to an approximate 10% increase in the value of SD2 for 2% ectopic beats.

All fragmentation measures show a large sensitivity to ectopic noise. There is a considerable increase in the variability of fragmentation measures with an increase in the percentage of ectopic noise. IALS is the least sensitive to noise with only a 2.5% RHRV with 2% of added ectopic beats. PAS, PIP, and PSS show an 11%, 242%, and 404% change in the HRV measures with the addition of 2% ectopic noise. These results suggest that fragmentation measures can be a good indicator of the presence of ectopic noise. Overall, the Gaussian noise affects the fragmentation measures less severely than the ectopic noise. The RHRV values for 10 dB Gaussian noise IALS, PAS, PSS and PIP are 1.6%, 41%, 154% and 158% respectively. The results show considerable variation in the relative change in HRV measures by the addition of an increasing percentage of ectopic noise.

## Conclusion

A novel preprocessing two-step method was developed to eliminate both technical and ectopic noise types for a comprehensive denoising approach. The tool was able to eliminate both types of noise without distorting the original signal with the performance indicators showing a high correlation and a low RMSE value. Overall, the relative percentage change in HRV measures observed was greater for ectopic noise than technical noise. This study, therefore, reaffirms that the majority of the HRV measures are affected by noise. However, the considerable variation in the sensitivity of HRV measures against the type and level of noise suggests the different contexts of preprocessing requirements for different HRV indices based on their robustness to noise should be considered when selecting HRV features to describe physiological signals.

## Data Availability

The data collected in this study is available upon request from the corresponding author.

## Code availability

The code developed in this study is available upon request from the corresponding author.
